# Long‐term effects of an intervention for families with parental severe mental illness—A 2.5‐year RCT follow‐up

**DOI:** 10.1002/jcv2.70178

**Published:** 2026-10-21

**Authors:** Anne D. Müller, Ida C. T. Gjøde, Mala Moszkowicz, Emma P. Frostholm, Johan S. Kristensen, Julie Forman, Merete Nordentoft, Anne A. E. Thorup

**Affiliations:** ^1^ Child and Adolescent Mental Health Center Copenhagen University Hospital – Bispebjerg and Frederiksberg Copenhagen Denmark; ^2^ Section of Biostatistics Department of Public Health University of Copenhagen Copenhagen Denmark; ^3^ Copenhagen Research Center for Mental Health – CORE Bispebjerg and Frederiksberg Hospital Copenhagen Denmark; ^4^ Department of Clinical Medicine Faculty of Health and Medical Sciences University of Copenhagen Copenhagen Denmark

**Keywords:** children of parents with mental illness (COPMI), early intervention, familial high risk, family‐based intervention, preventive psychiatry, randomized controlled trial

## Abstract

**Background:**

Children of parents with severe mental illness are at markedly increased risk of developing psychiatric disorders themselves. Interventions that target family‐related risk and protective factors may represent a promising approach in preventive psychiatry. This study evaluated the long‐term effects of the VIA Family intervention, a family‐based program designed for children at familial risk of severe mental illness and their families.

**Methods:**

We conducted a 2.5‐year follow‐up of a randomized clinical trial comparing the VIA Family intervention with treatment as usual (TAU). Eligible participants were families with children aged 6–12 years at baseline and at least one biological parent with a current or past severe mental illness. Families in the intervention arm participated in VIA Family for 1.5 years. The primary outcome was child mental health, assessed with the Strengths and Difficulties Questionnaire (SDQ). Secondary outcomes were child global functioning and the quality of the home environment (support and stimulation). Trial registration: clinicaltrials.gov (NCT06218693).

**Results:**

At follow‐up, 65 out of 95 families (68% retention) participated, including 77 children. The estimated treatment difference for SDQ was −0.23 (95% CI: −3.11 to 2.66), indicating no measurable long‐term impact of the intervention on child mental health compared to TAU. No significant differences emerged between intervention and TAU groups for the secondary outcomes, changes in global functioning and home environment quality.

**Conclusion:**

Our findings highlight the challenges of achieving significant and specific impacts with interventions for families with children at familial risk for the development of mental illnesses. The results emphasize the need for further research to optimize family‐based programs for familial high‐risk populations.

## Key Points

**What's known?**

- Children of parents with severe mental illness have elevated risks for psychiatric disorders. Family‐based interventions aim to reduce children's long‐term risk and strengthen protective factors.

**What's new?**

- This 2.5‐year follow‐up of the VIA Family randomized clinical trial found no significant differences between a family‐based intervention and treatment as usual on child mental health, global functioning, or home environment.

**What's relevant?**

- Results highlight the difficulty of achieving measurable benefits in heterogeneous high‐risk families, where effects may be subtle, context dependent, or not captured by standard measures. Preventive psychiatry is not “one‐size‐fits‐all”; future research should identify what works for whom through stratified designs, mixed‐methods outcomes, and larger, longer‐term studies.

## Graphical abstract

This 2.5‐year follow‐up of the VIA Family trial found no significant long‐term differences between a family‐based preventive program and treatment as usual in child mental health, global functioning, or home environment. Results emphasize the difficulty of achieving lasting benefits in heterogeneous high‐risk families.

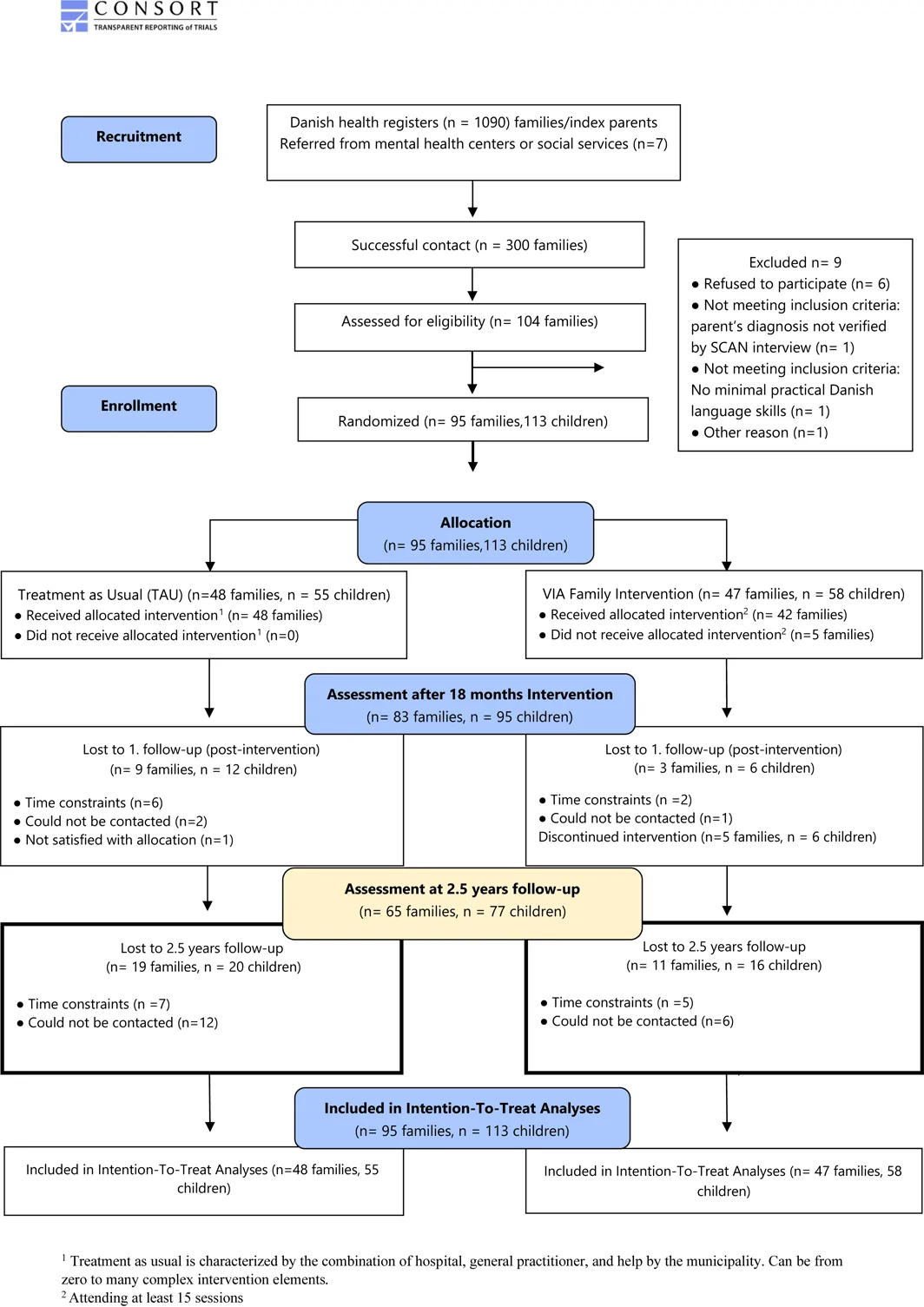

## INTRODUCTION AND AIMS

The transmission of mental illness across generations is not inevitable. Targeted, family‐based interventions offer a promising way to disrupt intergenerational pathways of risk—but their long‐term impact remains insufficiently understood. Globally, the prevalence of patients in mental health treatment who are also parents ranges from 12.2% to 45.0%, depending on definitions and diagnostic criteria used (Maybery & Reupert, 2018). In Denmark, 12.8% of children under age 16 live with a parent who has received psychiatric treatment for a mental illness (Christesen et al., 2022).

Parental mental illness profoundly impacts not only the individual with the illness but also their closest relatives, that is, their family and children. These effects often manifest as increased family and parental stress, reduced social support, and economic instability (Pierce et al., 2020), creating a non‐optimal environment for the children. Consequently, children of parents with a mental illness more often experience early signs of vulnerability such as mental and physical health problems, socioeconomic adversities, and childhood trauma compared with their peers (Brandt et al., 2022; Pierce et al., 2020; Sandstrom et al., 2019). Genetic heritability, combined with psychosocial stressors, lead to an imbalance of risk and protective factors during development, increasing the likelihood of mental health problems later in life (Uher et al., 2023). Despite the elevated risk for lifetime mental illness, not all children of parents with mental illness will develop mental health problems. Families with parental mental illness are diverse; they face various difficulties and may experience different needs for support.

In response to this, multi‐component family‐based interventions have been developed with the purpose of supporting children at familial risk and their families and to promote resilience (Hosman et al., 2009; Moltrecht et al., 2024; Puchol‐Martínez et al., 2023). Family‐based interventions propose promising potential in reducing risk factors and enhancing protective factors at a family level, potentially lowering familial high‐risk children's risk of mental illness or other negative life outcomes in adulthood (Lannes et al., 2021; McGovern et al., 2024). However, a critical gap in the literature exists regarding the long‐term effectiveness of these interventions, particularly in heterogenous populations (Duffy et al., 2023; McGovern et al., 2024; Moltrecht et al., 2024; Puchol‐Martínez et al., 2023). Most studies assess outcomes within 12–24 months post‐intervention, leaving the durability of effects over time largely unknown (Moltrecht et al., 2024; Puchol‐Martínez et al., 2023). Therefore, long‐term follow‐up studies are essential to understanding whether family‐based interventions yield sustainable improvements in child and family outcomes.

This study aims to address that gap by evaluating the long‐term impact of the VIA Family intervention, a family‐based intervention designed for families in which a parent has a history of severe mental illness (SMI), that is, recurrent major depression, bipolar disorder, or schizophrenia spectrum disorder (Duffy et al., 2023; Müller et al., 2019). Initial findings showed no significant effects at the end of the 18‐month implementation period (Gjøde et al., 2024; Müller et al., 2024a, 2024b). However, the intervention focused on strengthening protective family processes, for example, family communication about mental illness, parenting strategies, and identification of a social support system for the family, which may take longer to manifest measurable change.

The present study evaluates whether the VIA Family intervention yields long‐term benefits 2.5 years after the end of intervention assessment. We hypothesized that families, that is, children and parents, allocated to the experimental intervention group would demonstrate better outcomes compared to controls, including greater reductions in children's mental health symptoms at follow‐up.

## MATERIALS AND METHODS

### Design

The current follow‐up study to a two‐armed, parallel‐group randomized trial was conducted 2.5 years after the end of the intervention. A detailed description of the design, the recruitment process, and the intervention components of the original trial was previously published (Müller et al., 2019). The current follow‐up study was registered at clinicaltrials.gov (registration number NCT06218693) and the original trial was registered at clinicaltrials.gov (registration number NCT03497663). All participants (>age 15) provided written consent for their participation. Legal guardians provided consent for their children's participation. The Danish National Ethics Board approved the trial (Approval number H‐17000450).

### Participants

For the original trial, eligible participants were families with (a) children aged 6–12 years, (b) at least one biological parent in treatment for SMI during their child's life, and (c) living in Copenhagen, Denmark. We excluded families at baseline with insufficient Danish language proficiency to participate in the intervention or assessment interviews, and families who had received intensive support in the primary sector regarding parental mental illness within 6 months before study entry (Müller et al., 2019). At baseline, we assessed child mental disorders as research diagnoses using the semi‐structured interview K‐SADS‐PL (Kaufman et al., 1997), administered by trained psychologists and confirmed at expert clinical conferences. Eligibility for the trial did not depend on the child's diagnostic status.

For the 2.5‐year follow‐up, we invited all baseline participants for assessment, excluding those who had opted out at the post‐intervention assessment, primarily due to time‐management constraints (*N* = 11) (see Figure 1 CONSORT flow diagram). To ease time constraints, we only invited one parent (selected by the parents themselves) from the baseline assessment to participate in the follow‐up study, contrary to both parents in the original trial (Müller et al., 2019). All families from the trial, regardless of dropout status, were included in the intention‐to‐treat analyses.

**FIGURE 1:**
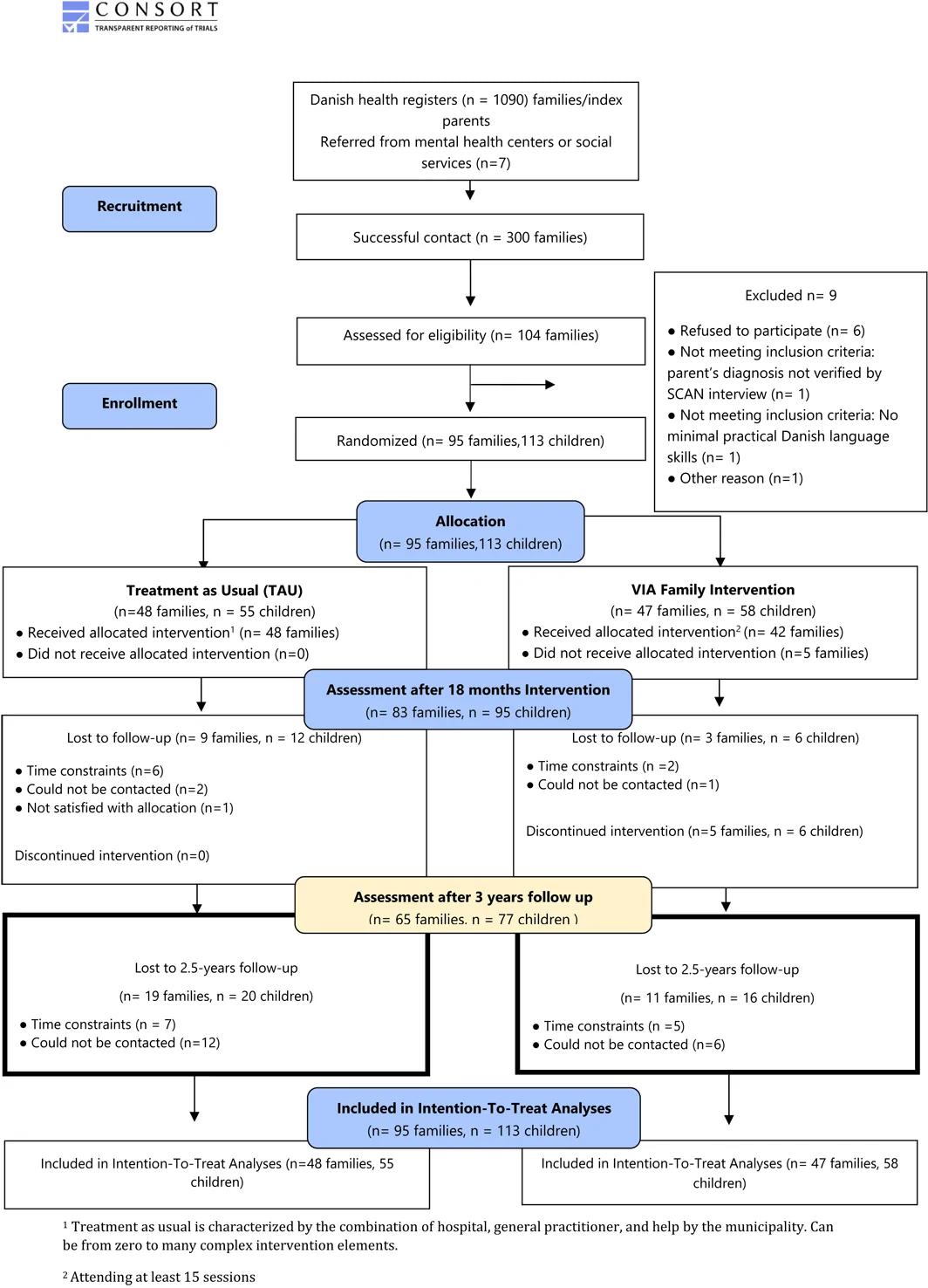
CONSORT flow diagram showing recruitment, allocation, baseline assessment, first follow‐up (post‐intervention), second follow‐up (2.5 years), and inclusion in ITT analyses.

### Randomization and masking

Families were randomly allocated (1:1) to either the experimental intervention VIA Family or treatment as usual (TAU) stratified by parental SMI diagnoses, using the secure, web‐based data capture system REDCap (Harris et al., 2009).

### Interventions and fidelity

TAU consisted of a short, standardized summary of all mental health support for children and families, and next‐of‐kin support options available within usual care in the social services, the mental health services in the Capital Region in Denmark, and relevant nongovernmental organizations.

The VIA Family intervention was designed as a family‐based multicomponent intervention over an 18‐month period. The intervention comprised case‐management, parenting support based on the Positive Parenting Program (Sanders, 2008), family‐based psychoeducation, social and legal guidance, peer support groups for children and adolescents, and support addressing early psychiatric conditions in children. The multidisciplinary team consisted of a social worker, a mental health nurse, a psychologist, and a family therapist. A child and adolescent psychiatrist (Anne A. E. Thorup) was designated as a consultant to participate in regular conferences with the team. The intervention was designed as a flexible and individually tailored program, allowing families to select modules based on their needs and motivations together with the case manager.

See prior publications for a detailed description of the interventions (Gjøde et al., 2024; Müller et al., 2019, 2024a, 2024b).

### Measures

For the current follow‐up study, we collected data between January 2022 and April 2024. Clinically trained assessors, blinded to group allocation, conducted assessments in families' homes, by phone/online, or at off‐clinic research facilities, according to family preference. Clinician‐rated outcomes were collectively rated under the supervision of a child and adolescent psychiatrist (Mala Moszkowicz).

The primary outcome for the current follow‐up study was the change in parent‐reported children's behavioral/mental health strengths and difficulties measured with the Strengths and Difficulties Questionnaire (SDQ) (Obel et al., 2003) from baseline to 2.5‐year follow‐up, whereas Children's Global Assessment Scale (CGAS (Shaffer, 1983)) was the primary outcome of the original trial. A low score on SDQ signifies low level of reported behavioral/mental health difficulties. Secondary outcomes were changes in children's global functioning measured with the CGAS and changes in stimulation and support in the home environment measured with the Home Observation for Measurement of the Environment (HOME) Inventory (Caldwell & Bradley, 2003). CGAS measures the lowest level of a child's psychosocial functioning over the past 30 days across home, school, and leisure time contexts, based on clinical interviews with both the child and parent. The HOME Inventory assesses the quality of the home environment through interviews and observation focusing on stimulating environments, supportive relationships, responsive parenting, and age‐appropriate resources. Higher scores on the CGAS reflect better overall functioning, whereas higher scores on the HOME Inventory indicate a more supportive home environment.

Exploratory outcomes included changes in both child‐ and parent‐reported measures. Children's self‐esteem was assessed using the self‐rated This Is Me (TIM) questionnaire, where higher scores reflect greater self‐esteem (Ouvinen‐Birgerstam, 2006). Parent outcomes encompassed changes in several domains; Global functioning was measured with the clinician‐rated interview Personal and Social Performance Scale (PSP) (Morosini et al., 2000), with higher scores indicating better functioning. Parental stress was evaluated using the parental stress subscale from the Parental Stress Scale (PSS) questionnaire (Berry & Jones, 1995; Nielsen et al., 2020) where lower scores reflect lower levels of perceived stress. Family functioning was assessed using the general family functioning subscale from the Family Assessment Device (FAD) questionnaire (Epstein et al., 1983), with lower scores indicating more positive family functioning. Lastly, dysfunctional parenting practices were evaluated using the Parenting Scale (PS) questionnaire (Arnold et al., 1993), with higher scores indicating more dysfunctional parenting. If legally or ethically warranted following the assessment, we submitted a notice report to social services regarding the participant's well‐being and need for support.

### Statistical analyses

All analyses were carried out in accordance with a pre‐published Statistical Analysis Plan (SAP) (Müller et al., 2024a, 2024b). In line with the SAP, SDQ was treated as the primary outcome in the present analyses. The primary and secondary outcomes were evaluated in the Intention‐To‐Treat population, including participants as randomized regardless of adherence to the intervention and study discontinuation. The effects of the intervention were estimated using a linear mixed model including randomization group and outcome at baseline as fixed effects and family ID as a random effect. Analyses of child outcomes further included child's sex, age, and short‐term memory scores as fixed effects, while analyses of parental outcomes further included sex and SMI diagnosis at baseline as fixed effects. Missing data were handled using 100‐fold multiple imputations that were iterated 20 times until convergence. Sensitivity analyses were performed (1) excluding outliers, (2) in the per protocol population, (3) without adjustment for baseline covariates, and (4) with adjustment for potential post randomization confounders related to differential drop out. Subgroup analyses examined potential differences in treatment effects by child baseline neurodiversity, age group, family functioning, degree of family participation, or primary motivation for participation. All analyses were performed with R statistical software version 4.5.0 (R Core Team, 2022) using the packages “Amelia” (Honaker et al., 2011), “mice” (Buuren & Groothuis‐Oudshoorn, 2011), and “lme4” (Bates et al., 2015).

## RESULTS

The mean interval from the post‐intervention assessment to the follow‐up assessment was 2.7 years (range 2.5–3.1 years). Thirty‐six families (out of 47, i.e., 77%) from the VIA Family group and 29 families (out of 48, i.e., 60%) from the TAU group participated in the follow‐up study (see Figure 1). Among participating families, 26 families in the VIA Family group (72%) and 25 families in the TAU group (86%) reported seeking mental health, school‐related, or social support for a family member between post‐intervention and long‐term follow‐up. Table 1 compares the baseline characteristics of families participating in the follow‐up visit with the non‐participants in each randomization group. Children from the TAU group who participated in follow‐up were more often female and more frequently had a mental illness diagnosis at baseline compared with those from the VIA Family group who participated in follow‐up. At baseline, families who participated in the follow‐up—compared with those in either allocation group who did not—were more likely to have mothers as the primary informants on child outcomes, to include children with a mental illness diagnosis, and to have a history of receiving family support.

**TABLE 1:** Baseline characteristics of families who participated in the follow‐up compared to non‐participants, and by allocation group (VIA Family vs. TAU).

Domain	Instrument	Treatment as usual		VIA Family		All
		*N* = 55 children		*N* = 58 children		
Children participants at 2.5‐year follow‐up	*n* (%)	Participating 35 (64)	Dropout 20 (36)	Participating 42 (72)	Dropout 16 (28)	113
Children's characteristics						
Biological sex, female	*n* (%)	18 (52)	10 (50)	16 (38)	9 (56)	53 (46.9)
Age at baseline	Mean (SD)	9.8 (2.1)	9.6 (1.9)	9.5 (2.1)	10.6 (1.3)	9.74 (2.0)
Global functioning	CGAS, mean (SD)	64.1 (15.3)	63.2 (15.4)	62.9 (13.1)	72.6 (11.5)	64.67 (14.2)
Any diagnosis^a^	K‐SADS‐PL, *n* (%)	14 (40)	7 (35)	12 (29)	3 (19)	36 (31.9)
Dimensional psychopathology	CBCL total problem score, T‐score (Danish norms), mean (SD)	59.77 (17.3)	65.2 (17.0)	59.0 (13.8)	49.9 (5.5)	59.00 (15.2)
High (≥15%) absence from school	Data from municipalities or schools, *n* (%)	4 (11)	2 (10)	3 (7)	1 (6)	10 (9)
Estimate of general intelligence	RIST, scaled scores, mean (SD)	102.2 (10.6)	97.6 (14.8)	101.8 (15.3)	106.9 (8.6)	101.96 (13.2)
General executive functioning	BRIEF, T‐score (Danish norms), mean (SD)	52.9 (12.4)	53.6 (11.9)	51.9 (12.6)	49.2 (9.9)	52.14 (12.0)
Social responsiveness	SRS‐2, T‐score, mean (SD)	57.3 (2.7)	59.9 (8.2)	58.7 (5.8)	57.3 (4.8)	58.2 (5.3)
Family, home, and parent characteristics						
Home environment and support	*N* (%)	29 (60)	19 (40)	36 (77)	11 (23)	95 (100)
Quality of home environment^b^	MC‐HOME/EA‐HOME, mean (SD)	46.4 (6.7)	47.4 (4.3)	46.3 (6.6)	46.8 (4.6)	46.6 (5.9)
Family receiving support from municipalities/MHS 6 months prior to baseline	Interview/Data from municipalities, *n* (%)	6 (20)	6 (32)	7 (19)	4 (36)	23 (24.2)
Index parent	*N* (%)	29 (60)	19 (40)	36 (77)	11 (23)	95 (100)
Lifetime^c^ Schizophrenia spectrum disorder	SCAN interview/patient journal, *n* (%)	5 (17)	1 (5)	6 (17)	0 (0)	12 (13)
Lifetime^c^ Bipolar disorder	SCAN interview/patient journal, *n* (%)	3 (10)	10 (53)	11 (31)	2 (18)	26 (27)
Lifetime^c^ Recurrent major and moderate depression	SCAN interview/patient journal, *n* (%)	21 (72)	8 (42)	19 (53)	9 (82)	57 (60)
Index parent with comorbid diagnosis^c^	SCAN interview/patient journal, *n* (%)	11 (38)	7 (37)	11 (31)	6 (55)	35 (37)
Primary caregiver	*N* (%)	29 (60)	19 (40)	29 (62)	18 (38)	95 (100)
Female	Interview, *n* (%)	26 (90)	15 (79)	25 (86)	12 (67)	78 (82)
Age	Interview, mean years (SD)	40.8 (6.4)	40.5 (6.8)	41.2 (6.1)	41.2 (3.1)	40.9 (5.8)
SMI or/and substance misuse lifetime diagnoses^d^	SCAN interview/patient journal, *n* (%)	20 (69)	15 (79)	21 (72)	13 (72)	68 (71.6)
General functioning (higher score better functioning)	PSP, mean (SD)	67.2 (17.3)	71.53 (14.7)	66.8 (14.3)	68.0 (15.5)	67.68 (15.5)
Parental stress (higher score more stress)	PSS, parental stress subscale, mean (SD)	3.10 (0.52)	3.42 (0.66)	2.95 (0.46)	2.73 (0.66)	3.06 (0.58)
Family functioning (lower score better functioning)	FAD General, mean (SD)	1.85 (0.43)	1.97 (0.59)	1.80 (0.57)	1.82 (0.64)	1.85 (0.55)
Employment status and educational level (*primary caregiver*)	*N* (%)	29 (60)	19 (40)	29 (62)	18 (38)	95 (100)
Currently employed or studying^e^	Interview, *n* (%)	23 (79)	16 (84)	26 (90)	15 (83)	79 (83.2)
Primary/lower secondary	*n* (%)	2 (7)	2 (10)	7 (24)	2 (11)	13 (13.7)
Upper secondary, vocational, or short‐cycle tertiary	*n* (%)	7 (24)	6 (32)	7 (24)	5 (28)	25 (26.3)
Bachelor's degree, equivalent, or higher	*n* (%)	20 (69)	11 (58)	15 (52)	11 (61)	56 (58.9)

Abbreviations: ADHD‐RS, Attention Deficit Hyperactivity Disorder Rating Scale (DuPaul et al., 1998); BRIEF, Behavior Rating Inventory of Executive Function (Gioia et al., 2000); CALS, Children's Affective Lability Scale (Gerson et al., 1996); CBCL, Child Behavior Checklist (Achenbach, 2001); CGAS, Children's Global Assessment Scale (Shaffer, 1983); CTS, Childhood Trauma Screener (Grabe et al., 2012); FAD, Family Assessment Device (Epstein et al., 1983), HOME, Home Observation for Measurement of the Environment (Caldwell & Bradley, 2003); K‐SADS‐PL, Kiddie Schedule for Affective Disorders and Schizophrenia‐Present and Lifetime (Kaufman et al., 1997); PSP, Personal and Social Performance Scale (Michalos, 2014); PSS, Parental Stress Scale (Berry & Jones, 1995); RCFT, Rey Complex Figure Test (Meyers & Meyers, 1995); RIST, Reynolds Intellectual Screening Test (Reynolds & Kamphaus, 2003); SCAN, Schedules for Clinical Assessment in Neuropsychiatry (Wing, 1990); SRS‐2, Social Responsiveness Scale‐Version 2 (Constantino & Gruber, 2012); TOMAL‐II, Test of Memory and Learning, 2nd Edition (Reynolds & Voress, 2007); WISC‐IV, Wechsler Intelligence Scale for Children, 4th Edition (Wechsler, 2003).

^a^ Current diagnosis within the last 8 weeks, excl. Elimination Disorder, Simple Phobia, Tics/Tourette's Syndrome.

^b^ For children living 50/50 with mother and father, the HOME interview was done in both homes.

^c^ During child's life (including the prenatal period).

^d^ Personality disorder, substance misuse, ADHD, PTSD, or eating disorder.

^e^ Any form of employment, no minimum working hours, absence from work due to for example, illness or maternity leave included as work.

Table 2 shows the estimated treatment differences for VIA Family compared with TAU at 2.5‐year follow‐up. No significant treatment effects were found for any of the child‐, parent‐ or family‐related outcomes. The estimated treatment differences were −0.23 (95% CI: −3.11 to 2.66) in SDQ; 2.73 (95% CI: −4.02 to 9.49) in CGAS, and 0.81 (95% CI: −3.1 to 4.71) in HOME. Results remained similar with and without adjustment for baseline covariates. Exploratory analyses, likewise, showed no differences in any prespecified outcome. Sensitivity analyses showed no noteworthy differences in results when including further baseline characteristics that differed between the groups as covariates, when excluding outliers from the analysis, and when restricting the analysis to the per protocol population. None of the subgroup analyses revealed significant differences between allocation groups on either the primary or secondary outcomes (see Figures [Link], [Link], [Link]).

**TABLE 2:** Estimated effects of VIA Family versus TAU on primary and secondary outcomes at follow‐up.

Outcome	Instrument	TAU	VIA Family	Estimated treatment differences^a^ (95% CI) VIA Family versus TAU	*p*
		Mean (SD)	Mean (SD)		
Children outcomes, *N* = 113					
Change in mental health: Strengths and difficulties	Strength and Difficulties Questionnaire (SDQ)‐Danish version (Obel et al., 2003). Parent rated (Danish norms) (Arnfred et al., 2019) Higher score: More difficulties	9.80 (7.53)	8.02 (5.83)	−0.23 (−3.11; 2.66)^a^	0.88
Change in children's global functioning	Children's Global Assessment Scale (CGAS) (Shaffer, 1983) Clinician rated Higher score: Better functioning	67.17 (17.71)	69.86 (14.10)	2.73 (−4.02; 9.49)^a^	0.42
Change in self‐esteem	This Is Me (TIM) self‐rated (Ouvinen‐Birgerstam, 2006) Higher score: More self‐esteem	5.08 (1.95)	5.06 (1.95)	−0.85 (−2.04; 0.34)^a^	0.16
Family outcomes, *N = 95*					
Level of stimuli and support in the child's home environment	The Home Observation for Measurement of the Environment (HOME) (Caldwell & Bradley, 2003) Higher score: Higher level of support	44.92 (5.24)	44.14 (8.10)	0.81 (−3.1; 4.71)^b^	0.68
Parent outcomes, *N* = 95					
Global functioning	Personal and Social Performance Scale (PSP) (Morosini et al., 2000) Higher score: Better functioning	69.3 (16.3)	69.1 (19.1)	−0.49 (−6.38; 5.39)^b^	0.87
Parental stress	Parental Stress Scale (PSS)–Parental Stress subscale (Berry & Jones, 1995; Nielsen et al., 2020) Lower score: Lower perceived parental stress	2.63 (0.73)	2.54 (0.77)	−0.001 (−0.24; 0.23)^b^	0.99
Family functioning	Family Assessment Device (FAD)–General Family Functioning (Epstein et al., 1983) Lower score: Better functioning	1.93 (0.44)	1.87 (0.49)	0.03 (−0.15; 0.21)^b^	0.75
Parent outcomes *N* = 113					
Dysfunctional parenting practices	Parenting Scale (PS) (Arnold et al., 1993) High score: Dysfunctional parenting	3.07 (06.55)	2.91 (0.62)	0.06 (−0.24; 0.36)^b^	0.68

*Note*: Estimated treatment differences are baseline‐adjusted mean difference in change based on a linear mixed model including family ID as random effect and with missing data replaced by multiple imputations.

^a^ Further adjusted for child's age, sex, and TOMAL at baseline.

^b^ Further adjusted for parents gender and severe mental illness diagnosis at baseline.

## DISCUSSION

This study evaluated the long‐term effects of a family‐based intervention, VIA Family, for children with elevated familial risk of severe mental illness. At the 2.5‐year post‐intervention follow‐up, there were no significant differences in the primary outcome—change in child mental health (measured with the SDQ)—or in any other outcomes between the VIA Family and TAU groups. These results resemble the non‐significant findings from baseline to end of intervention (1.5 years after inclusion) in the original trial (Gjøde et al., 2024; Müller et al., 2024a, 2024b). Contrary to our hypothesis, there were no significant differences favoring VIA Family at long‐term follow‐up, but also no indications of negative effects.

Our findings are consistent with previous studies that have failed to demonstrate the long‐term effects of interventions for children of parents with severe mental illness (Landi et al., 2025; Moltrecht et al., 2024; Puchol‐Martínez et al., 2023). An exception is the long‐term follow‐up of a trial conducted by Compas et al. (2015), which evaluated a family‐based cognitive‐behavioral group intervention for families with a parental history of depression and found positive outcomes on children's symptoms of mental illness at a 24‐month follow‐up.

One explanation of the null findings of the current study is that the experimental intervention, VIA Family, did not produce measurable superior effects on the pre‐specified outcomes. This could also explain the absence of changes at the first follow‐up (at the end of intervention) (Gjøde et al., 2024; Müller et al., 2024a, 2024b). Nevertheless, qualitative findings from interviews with families participating in VIA Family at the end of intervention revealed that families participating in the intervention did perceive meaningful changes based on their perceived needs for support (Müller et al., 2025). Therefore, the absence of significant effects in this study also invites a closer examination of methodological factors of evaluating family‐based interventions in real‐world settings, particularly regarding sample heterogeneity, sample size, intervention variability, and the sensitivity of outcome measures that may have influenced the results.

VIA Family was intentionally designed as a flexible, multicomponent intervention framework rather than a rigidly standardized program. Accordingly, the trial evaluates the effectiveness of offering this structured yet individually tailored model of support as implemented in practice. Families in the trial differed substantially in baseline characteristics, for example, child's mental health status, socioeconomic conditions and perceived needs for support (Gjøde et al., 2024; Müller et al., 2024a, 2024b). Although recruitment was based on familial high‐risk status, the cohort included children with and without pediatric mental health problems and neurodiversity at baseline. Hence, the intervention spanned both early‐intervention and preventive elements at the individual level, which should be considered when interpreting the findings. As a result, families in both groups received highly individualized support based on their preferences and needs. While such flexibility reflects the realities of clinical practice, an intentional feature of the multicomponent design of the VIA Family intervention, and is central to the rationale for pragmatic trials, it complicates interpretation: different families received different “doses” and types of support, making it difficult to assess group‐level effects (Giraudeau et al., 2022). This variability may dilute any average treatment effect. In complex, multi‐component interventions, such as VIA Family, some families may experience meaningful improvements while others do not, yielding null results overall. Service use during the follow‐up period was common in both groups, however, more TAU families reported seeking support. This greater use of support in the control group may have reduced between‐group differences over time and could therefore partly explain the absence of significant intervention effects at follow‐up. Although we explored potential subgroup effects, the trial was not sufficiently powered to detect small‐to‐moderate differences between groups. Moreover, the diversity of risk factors, including differences in environmental and genetic vulnerabilities (e.g., a child having two parents with SMI), further reduces the likelihood of consistent effects across the full sample. Finally, it is possible that changes did occur, but in areas not captured by the selected quantitative measures. Prior research suggests that family‐based interventions may improve facets of family cohesion, communication, and parent‐child relationships, domains not always captured fully in standardized tools like the SDQ, CGAS, or HOME. Thus, the absence of significant effects may reflect both true null findings and limitations in what and how we measured change.

### Strengths and limitations

This study offers several important strengths but also includes methodological and contextual limitations that should be considered when interpreting the results. A key strength is the long‐term follow‐up, which includes blinded assessments and carefully collected clinical data of high quality. However, the study is limited by sample size and attrition. Although 65 families (68% of the baseline sample) participated in the follow‐up, this may not have been sufficient to reliably detect long‐term or subtle effects. The relatively small sample size of the original trial (95 families) reduces the statistical power to assess the effects of effect heterogeneity in this diverse population. Moreover, potential differential dropout introduces a risk of bias in the estimated treatment effects, particularly if data are not missing at random, which is a limitation that cannot be fully addressed by multiple imputations. Furthermore, in this pragmatic trial, the use of TAU as comparison likely reduced detectable between‐group differences, as families were free to access available support. Additional intervention effects would therefore be expected to be modest in a small, clinically heterogeneous sample. Additionally, the follow‐up study and the SAP were registered after data collection had commenced. Although analyses followed a pre‐written SAP, delayed registration represents a limitation in terms of prospective transparency and limits the ability to independently verify prespecifications. A further limitation is that CGAS was classified as a primary outcome in the original trial documents, whereas SDQ was treated as the primary outcome in the present follow‐up analysis. This discrepancy should be considered when interpreting the findings. Another limitation concerns measurement. While we assessed child‐reported global functioning, self‐esteem, and perceived support at home, we did not include child‐reported mental health outcomes, for example, child‐reported SDQ. Previous research suggests that child‐reported data may reveal stronger effects than parent‐report alone, especially for internalizing symptoms (Lannes et al., 2021). Moreover, although VIA Family targeted specific family processes, the primary outcomes were broad composite measures, which may have limited sensitivity to detect more specific changes in targeted domains. Adverse effects were not assessed using a standardized measure at long‐term follow‐up, and conclusions regarding long‐term negative effects should therefore be interpreted with caution. Finally, recruitment and timing may have affected families' engagement with the intervention. Participants were primarily recruited via the Danish national patient registers, without regard to current service use or timing of parental illness. As a result, some families may not have experienced an immediate or pressing need for support—unlike those currently engaged with health or social services. This variation in perceived relevance or urgency may have affected motivation to participate, as intervention timing is known to influence engagement and outcomes (Finan et al., 2018). Lastly, the evolving needs of families over time may also have influenced the outcomes.

### Implications and future directions

The findings of this study highlight three key priorities for future research. First, the substantial heterogeneity in family characteristics and needs suggests that stratified or subgroup‐focused intervention designs may be necessary. Targeting more specific risk profiles could improve sensitivity to intervention effects that are otherwise diluted in diverse samples. Second, our results point to the importance of using mixed‐methods approaches, which integrate qualitative and quantitative methods and data, and more sensitive outcome measures. Integrating qualitative data and developing multidimensional metrics may provide a more comprehensive understanding of how family‐based interventions exert their effects. Finally, future trials should address statistical power and participant retention. Larger sample sizes and improved strategies for minimizing attrition are essential to reliably detect small‐to‐moderate effects and to support more definitive conclusions about the effectiveness of interventions for children of parents with SMI.

## CONCLUSION

Although this study did not find significant long‐term effects of the family‐based VIA Family intervention on child mental health or quality of home environment, it provides valuable insights into the challenges of assessing outcomes of family‐based interventions over a long time. The findings underscore the importance of tailoring interventions to specific subgroups and use of robust, multidimensional evaluation frameworks. Continuing research in this area is essential to refine intervention models, explore mechanisms of change, and ensure sustainable improvements in mental health outcomes for children and their families when a parent has a mental illness.

## AUTHOR CONTRIBUTIONS

**Anne D. Müller**: Conceptualization; data curation; formal analysis; writing—original draft; writing—review and editing. **Ida C. T. Gjøde:** Conceptualization; writing—review and editing. **Mala Moszkowicz**: Conceptualization; data curation; investigation; supervision; writing—review and editing. **Emma P. Frostholm**: Data curation; investigation; writing—review and editing. **Johan S. Kristensen**: Data curation; investigation; writing—review and editing. **Julie Forman**: Supervision; writing—review and editing. **Merete Nordentoft**: Conceptualization; writing—review and editing. **Anne A. E. Thorup**: Conceptualization; supervision; writing—review and editing. All authors have read and approved the final manuscript.

## CONFLICT OF INTEREST STATEMENT

The authors declare no conflicts of interest.

## ETHICAL CONSIDERATIONS

The VIA Family study was approved by the Danish national ethics board at the Committee on Health Research Ethics in the Capital Region of Denmark (Approval No. H‐17000450) on 20.07.2017. All participants ≥15 years have given written informed consent. For children younger than 15 years, written consent was obtained from their legal guardians.

## AI STATEMENT

The authors declare that they have not used AI in any capacity that would require disclosure in accordance with Wiley's AI Guidelines, nor in any capacity that would reasonably require discloser for editors, reviewers and readers to properly evaluate their research or manuscript. The authors confirm that AI has not been used for purposes including but NOT limited to drafting and editing, to generate substantial text or restructure arguments, nor in the research methodology. The authors take full responsibility for the accuracy of this statement.

## TRIAL REGISTRATION

The present follow‐up study has been registered on 11.04.2024 at ClinicalTrials.gov, identifier: NCT06218693; the original trial was registered at ClinicalTrials.gov under NCT03497663 at https://clinicaltrials.gov/study/NCT03497663.

## Supporting information

Figure S1

Figure S2

Figure S3

## Data Availability

The data that support the findings of this study are available on request from the corresponding author. The data are not publicly available due to privacy or ethical restrictions.
