# Supplementary material for: Long‐term effects of an intervention for families with parental severe mental illness—A 2.5‐year RCT follow‐up
Source: JCPP Adv. 2026 Oct 21:e70178. Online ahead of print. doi: 10.1002/jcv2.70178 (PMC13686432; doi:10.1002/jcv2.70178)
Supplement: Supplementary file 2 [file JCV2-9999-e70178-s002.pdf]

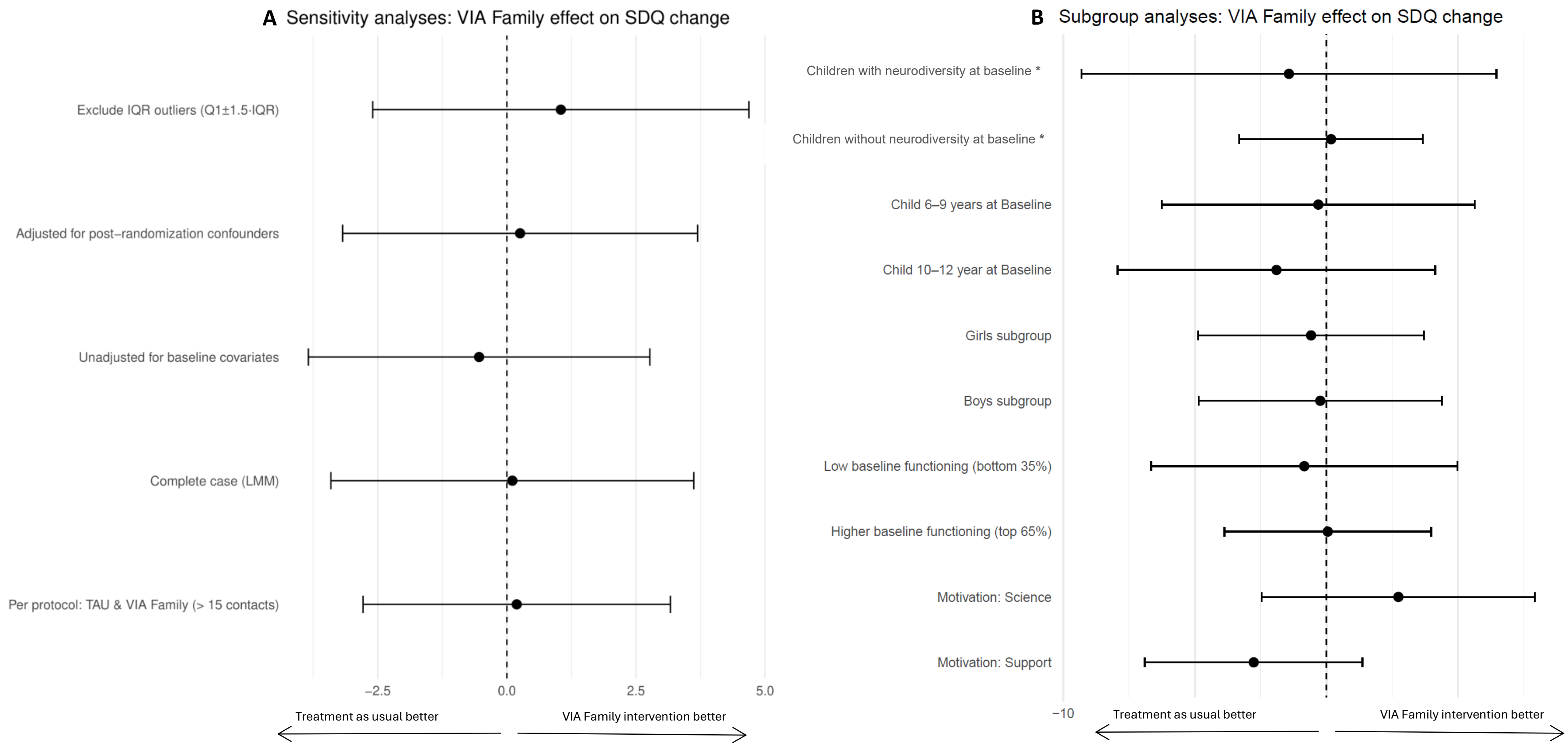

Figure S1. Forest plots of (A) sensitivity analyses and (B) subgroup analyses for the primary outcome Strengths and Difficulties Questionnaire (SDQ) total score. Estimated effects of VIA Family vs. Treatment as usual at 2.5-year follow-up with 95% confidence intervals. Analyses were performed in the intention-to-treat (ITT) population, except for the complete-case analysis.

* Neurodiversity = Attention-Deficit/Hyperactivity Disorder (ADHD) or Autism Spectrum Disorder (ASD) according to K-SADS diagnostic criteria.
